# Identification of Defensive Performance Factors in the 2010 FIFA World Cup South Africa

**DOI:** 10.3390/sports4040054

**Published:** 2016-11-30

**Authors:** Claudio Alberto Casal, Miguel Ángel Andujar, José Luis Losada, Toni Ardá, Rubén Maneiro

**Affiliations:** 1 Department of Science of Physical Activity and Sport, Catholic University of Valencia San Vte Mártir, 46900 Valencia, Spain; 2 Department of Physical and Sport Education, University of A Coruña, 15000 A Coruña, Spain; miguel.andujar@udc.es (M.Á.A.); ardsd@udc.es (T.A.); 3 Department of Methodology of Behavioral Sciences, University of Barcelona, 08000 Barcelona, Spain; jlosada@ub.es; 4 Department of Science of Physical Activity and Sport, Pontifical University of Salamanca, 37007 Salamanca, Spain; rmaneirodi@upsa.es

**Keywords:** elite soccer, defensive phase, observational methodology, performance analysis

## Abstract

The aim of this study was to determine the efficacy of defensive play in elite football, to identify variables associated with the direct recovery of ball possession, and to propose a model for predicting the success of defensive transitions. We analyzed 804 transitions in the final stages of the Fedération Internationale Football Association (FIFA) World Cup 2010, and investigated the following variables using univariate, bivariate, and multivariate analyses: duration of defensive transition, possession loss zone, position of players at the start and end of the defensive transitions, defensive organization, general defensive approach, time of the match, position of defense, zone in which the offensive transition ends, match status, and outcome of the defensive transition. We found that the defensive transitions started most frequently in the middle offensive zone (48.9%), with an organized defense set-up (98.8%), and were unsuccessful on 57.2% of occasions. The bivariate analysis showed that the variable most strongly associated with direct recovery of the possession of the ball (*p* = 0.018) is the area in which the ball is lost, and the multivariate analysis showed that the duration of the defensive transition can be used as a performance indicator, with transitions lasting between 0 and 15 s associated with a higher likelihood of directly recovering the ball. This work has allowed us to identify a pattern of tactical-strategic behavior with major probabilities of success in the defensive transitions. These results will be able to be used by coaches to improve the performance of their teams in this type of situation in the game.

## 1. Introduction

Soccer is a sport characterized by constant, dynamic interactions between the members of two teams [1]; these interactions change continuously throughout the game, and when an action executed by a team is successful, the team will try to repeat it and the opposing team will try to modify its behavior to prevent this from happening. Considering the complex, dynamic nature of soccer [2,3,4], reliable performance indicators are very difficult to identify, but this should not prevent research into strategic technical-tactical actions that may help to ensure or predict better performance.

Numerous performance indicators have been proposed in recent years [5], but they must be used carefully, as they can be influenced by some situational variables [6] such as match status, match location and quality of opposition. It is also important to check that the studies in which these indicators have been used have accounted for the interactions among the teams and players [7], the multifactorial structure [1,8,9] and the complex nature of soccer.

For instance, if the aim of a study is to produce results designed to improve defensive performance in competition soccer, the focus should be on defensive transitions, which are defined as a series of technical-tactical actions and strategies executed by a team after it has lost possession of the ball and while it is repositioning itself defensively and adopting a general defense strategy [10]. The importance of these transitions for the course of play has been noted by several authors [10,11,12]. Most goals and risk situations occur during these situations [9,13,14,15], and therefore the moment at which a team loses possession of the ball is critical in defense set-ups. Although most studies that have employed observational methodology to analyze performance in soccer have focused on attack situations [16], several studies, such as that by Martins [17], have shown that match status and the number of minutes played modify defensive play patterns and that position of defense lines in the defensive zone and defensive midfield and on the wings is the main defensive strategy used by teams. A recent study by Barreira et al. [18] indicated that direct recovery of the ball in central areas of the field increased attack efficiency and reported that protecting the midfield areas and pressurizing the player with the ball were key factors for improving the overall organization of the defense. Vogelbein et al. [19], in turn, reported that rapid recovery of ball possession was an important determinant of defensive performance. According to Casal [10] most balls are recovered by the defense line in the defensive midfield with an organized defense set-up.

In this study, we further explore the defensive phase in soccer with three objectives: to objectively show how elite soccer teams execute defensive transitions, to identify variables associated with the effectiveness of these transitions, and to build a model to predict their success. In order to achieve these aims, we have tried to sort out some of the main restrictions present in previous studies. This way, several high-level teams have been analyzed, not only one. The multifactorial character of the football has been taken into consideration, analyzing several performance factors and studying the influence of some on others in a multivariate way. Also the interactions between the teams and players were taken into account, analyzing the behavior of both teams in the interaction; it has been a question of controlling some of the situational variables that could influence the behavior of the teams, such as the match location, the match status and the quality of the opposition. The results of the work will allow coaches to understand the tactical-strategic behavior patters of the teams with major probabilities of success in this type of situation, with which they will be able to design training tasks to reproduce them in meetings and to improve the performance of their teams.

## 2. Method

### 2.1. Study Design

We employed a direct, non-participative, systematic, observational methodology design in a natural setting [20]. The level of participation is non-participative observation, given that the observer does not interact with the observed players and the degree of perceptivity is complete, direct observation. According to the nomenclature of Anguera and Mendo [21], the design was nomothetic (observation of several teams), follow-up (separate observation of each competing team in all match recordings), and multidimensional (various levels of response).

### 2.2. Participants

We analyzed 804 defensive transitions from eight matches (quarter-finals, semi-finals, and finals) of the FIFA World Cup in South Africa, 2010. Numerous situational variables were taken into account to select the sample and improve the generalizability of results by reducing variability [5,22]. These variables were match location (all the matches were played on neutral ground), match status, quality of opposition (top 16 teams in the World Cup), and competition phase (quarter finals, semi-finals, and final, where a draw is not allowed and therefore there is no room for speculation about the result). The unit of analysis was comprised between the moment in which the team observer lost the possession of the ball, until the opposition team ended its offensive phase. Only defensive transitions that started and ended directly, i.e., without interruptions of play [18] and control of the ball by the goalkeeper, were recorded and analyzed. The characteristics of the matches analyzed are summarized below (Table 1):

### 2.3. Observational Tool

We designed an ad hoc observation instrument with a field-format design, as described by Anguera et al. [23] for observational methodology studies (Table 2). The variables were chosen from some of the most recent studies in this field [1,5,6,7,10,17,18,19,24,25,26].

### 2.4. Procedure

Video recordings of public television broadcasts were used to analyze and code used the match data. Because the video recordings were public, confidentiality was not an issue and authorization was not required from the players observed or their representatives. Furthermore, the information cannot be considered either personal or intimate, as the research consisted solely of naturalistic observations in public places, and it was not anticipated that the recordings would be used in a manner that could cause personal harm [27].

The data were recorded using the software program NAC SPORT ELITE 42, and the notational analysis was made with LINCE [28]. Only defensive transitions in which the team directly lost possession of the ball were recorded. The binary logistic regression module in IBM SPSS Statistics 21 was used for data analysis.

The level of significance for each performance indicator was set at 5% as usual in comparable scientific studies [29].

Each observer was trained following the protocols described by Losada and Manolov [30]. To check the progress made by the observers in the use of the instrument, a set of data recorded at different moments by each observer was analyzed to study intraobserver reliability, with a minimum agreement level of 80% established [31].

To confirm the quality of the data, interobserver agreement was analyzed using the kappa statistic for each category (Time = 1; Loss of possession zone = 0.820; Position of players on initiation of defensive transition = 0.247; Defensive organization = 1; General defensive approach = 0.812; Position of defense lines = 1; Zone in which defensive transition ends = 0.881; Position of players at end of defensive transition = 1; Duration = 1; Match status = 1). The mean kappa value was 0.887, which corresponds to very good agreement according to the criteria of Fleiss, Levin and Paik [32].

In accordance with the objectives of this study, three complementary analyses were performed: a descriptive or univariate analysis, a comparative or bivariate analysis, and a predictive or multivariate analysis. The univariate analysis was used to determine how defensive transitions are executed in elite soccer. A bivariate analysis using the independent chi-square test was performed to identify possible predictors of success. The dependent (qualitative, dichotomous) variable had two possible outcomes: success (direct recovery of the ball without the intervention of the goalkeeper) and failure (shot or goal by other team, or interruption of play of any type). Finally, to build a model to explain the efficacy of defensive transitions in soccer, we performed logistic regression analysis, which is a statistical model used to investigate the association between a qualitative, dichotomous dependent variable (binary or binomial logistic regression) or a dependent variable with more than two outcomes (multinomial logistic regression) and one or more independent, qualitative or quantitative, predictor variables or co-variables. An exponential-type equation is applied initially, but this can be made linear through logarithmic transformation. The technique can be used to predict the outcome of defensive transitions and at the same time identify the variables that intervene in this outcome. The dependent variable was the outcome of the defensive transition (success vs. failure) and the predictor variables were the variables significantly associated with success in the bivariate model. In short, we built a binary logistic regression model to predict the outcome of defensive transitions in soccer in which our selection of study variables was guided by theory, followed by stepwise regression with application of the Wald test to determine the inclusion/elimination of variables.

## 3. Results

### 3.1. Descriptive Analysis

A total of 804 defensive transitions that started with a direct loss of possession were identified in the eight matches analyzed; this corresponds to an average of just over 100 ball losses per match and 50 per team. Of these defensive transitions, 66.3% lasted between 1 and 15 s. The transitions were initiated in the offensive midfield in 48.9% of cases and with the attacking line in front of the rear line of the opposing team in 43.7% of cases. Practically all the transitions were initiated with an organized defense set-up (98.8%). Overall, the teams adopted persistent defense tactics in 57.7% of cases. The defensive transition ended in the defensive midfield in 37.1% of cases, and the ball was recovered by the rear line in front of the attacking line of the opposing team in 48.4% of cases. The teams failed to directly recover the ball in 57.2% of cases (Table 3).

### 3.2. Bivariate Analysis

Table 4 shows the different predictors with their corresponding significance level in relation to the dependent variable (success of defensive transition). As can be seen, six variables were significantly associated with success: possession loss zone (ZP) (χ^2^ = 5.615, *p* = 0.018), position of defense lines (PS) (χ^2^ = 8.953, *p* = 0.003), zone in which defensive transition ends (ZF) (χ^2^ = 8.629, *p* = 0.003), position of players at end of defensive transition (CEIF) (χ^2^ = 8.121, *p* = 0.004), duration 0–15 (χ^2^ = 7.326, *p* = 0.007), and duration >30 s (χ^2^ = 8.101, *p* = 0.004).

### 3.3. Multivariate Analysis

The logistic regression analysis allowed us to define the final following statistical model:

Logit (Success) = β_0_ + β_1_(ZP) + β_2_(PS) + β_3_(ZF) + β_4_(CEIF) + β_5_(DJ1) + β_6_(DJ3) (1)

The results of the Hosmer-Lemoshow goodness of fit test (χ^2^ = 11.586, *p* = 0.171) were not significant (>0.05), indicating that the predicted model was not significantly different from the observed one.

The model correctly predicted 99 of the 344 successful defensive transitions; this corresponds to a sensitivity of 28.8%. Of the 460 unsuccessful transitions, 97 were correctly predicted, corresponding to a specificity of 78.9%. Overall (considering success and failure), the model correctly classified 57.5% of all defensive transitions. The sensitivity of the model holds more interest in this case than its specificity, as our goal was to identify factors that contribute to the success of defensive transitions in terms of recovering the ball.

The results of an omnibus test, performed to detect whether or not the variables selected were capable of predicting the dependent variable, showed that they were, as all the variables were significant.

We were also interested in investigating the extent to which the variables in the model were capable of predicting success (Nagelkerke R Square). The strength of prediction was very low, suggesting that while the predictors are theoretically good, they lack statistical power (R^2^ = 4.3%).

As shown in Table 5, only one variable (duration 0–15 s) was included in the final model (*p* = 0.022). The coefficient sign was negative (B = −0.208), indicating that the longer a defensive transition lasts, the less likely it is that the team will regain possession of the ball. The odds ratio (Exp(B)) indicates that a defensive transition lasting between 16 and 30 s has an 0.812-fold decreased likelihood of being successful. The other variables were not included in the model because their coefficients did not differ significantly from 0. In other words, they added nothing to the ability of the model to predict direct ball recovery.

Using the estimators, the equation can be expressed in logit units as follows:

Logit (RJ) = 2.017 − 0.208·DJ1
(2)

The following calculation was performed to estimate the likelihood of recovering the ball in the first 15 s:

100 × [Exp(β) − 1]   100 × [0.812 − 1] = −18.8%
(3)

The result indicates that when the defensive transition lasts longer than 15 s, the probability of directly recovering the ball drops by almost 20%.

We also analyzed the likelihood of directly recovering the ball in the first 15 s:

(4) $$P\;\left[\mathrm{success}\;\mathrm{DJ}1\right]=\frac{e^{\beta_{0}+\beta_{1}}}{1+e^{\beta_{0}+\beta_{1}}}$$

(5) $$P\;\left[\mathrm{success}\;\mathrm{DJ}1\right]=\frac{e^{2.017-0.208}}{1+e^{2.017-0.208}}=0.5518$$

The result indicates a likelihood of 55.18%. This is 12.38% higher than the actual percentage of direct recoveries observed in the matches (42.8%).

## 4. Discussion

The aim of this study was to describe how elite soccer teams execute defensive transitions directly following loss of the ball, to identify variables that were independently and significantly associated with direct recovery of possession and to propose a model for predicting the success of defensive transitions. Therefore, the present work has been able to describe the way in which elite soccer teams execute this type of play. It was also possible to identify several variables that are closely related to the direct recovery of possession and, therefore, can influence it. Finally, a model has been constructed that allows us to predict the result of the defensive transitions. Specifically, if the teams, after losing possession of the ball, put an immediate pressure on the rival team, in order to try to recover the ball quickly, the chances of success will be quite high (55.8%).

The findings indicate that direct possession is lost just over 100 times in each match, and that most defensive transitions (66.3%) last between 1 and 15 s. These data are similar to those of Vogelbein et al. [19], who reported that teams require between 10 and 14 s to directly recover the ball. They also coincide with those of Casal [24], who showed that offensive transitions that start with direct recovery of the ball last for an average of 16 s, which means that the duration of the corresponding defensive transitions would be the same. It is difficult to compare our results with those of similar studies, as only a few distinguish between defensive transitions that are started directly and those that are started indirectly.

A majority (48.9%) of the defensive transitions we analyzed started in the offensive midfield. This finding is comparable to reports by Barreira et al. [18], Casal [24], Lago-Ballesteros et al. [26], and Tenga et al. [7], in which most ball recoveries occur in the rear defensive zone and the offensive midfield, which logically means that the ball is lost in the same areas. These data can be explained by the greater density and defensive intensity of the teams in these areas when they are close to the goal. Consequently, almost half (43.7%) of the defensive transitions in our sample started when the attacking line was in front of the rear line of the opposing team. This indicates that on most occasions, the ball is lost with this same set-up. Similarly, Casal [24] showed that the defense recovered the ball when their rear line was positioned in front of the attacking line of the opposing team in 35.3% of cases. This indicates that both offensive and defensive transitions are initiated with this formation.

Practically all of the defensive transitions (98.8%) started with an organized defense set-up; this is consistent with the observation by Casal [24] that 81.3% of offensive transitions are initiated when the defending players are in position. When dealing with elite teams, they will always try to maintain a balance between the offensive and defensive phase, keeping certain players able to start the defensive phase immediately after losing possession of the ball.

On losing possession of the ball, the teams analyzed in this study adopted a persistent defensive strategy in 57.7% of cases, indicating the concern to quickly recover possession of the ball. The transition ended in the defensive midfield in 37.1% of cases, with the ball being recovered by the rear line in front of the attacking line of the opposing team on 48.4% of occasions. These data agree with those referring to the starting zone of defensive transitions for the same reason. There is a higher percentage of recoveries in this area because there is an increase of density and intensity of the defense due to the goal defense proximity. The teams failed to recover the ball directly in 57.2% of cases, which shows that in most transition cases, changes of possession are made with an interruption of the game, thus losing the opportunity to make a counter-attack to take advantage of the defensive reorganization of the opposing team.

The results of the bivariate analysis showed that successful recovery was significantly associated with six variables: possession loss zone, position of defense, zone in which the defensive transition ends, position of players when the defensive transition ends, and duration of the defensive transition. The strongest association was detected for the zone in which possession is lost, indicating that the area in which a team loses the ball and in which the defensive transition starts may influence the likelihood of direct recovery.

Finally, the performance of a logistic regression analysis has allowed us to test an integrated model which allows us to know how the different studied variables are combined when explaining or predicting the direct recovery of the possession of the ball. The obtained results allow us to conclude that the duration of the defensive phase is an indicator of the performance in this type of situation. Specifically, the results indicate that defensive transitions lasting between 0 and 15 s are more likely to result in direct recovery of the ball than longer transitions. Similarly, Vogelbein et al. [19] reported that higher-ranked teams in the German Bundesliga recovered the ball more rapidly than lower-ranked teams. These results are also consistent with those of numerous studies that indicate that successful teams hold onto the ball for longer than less successful teams [22,25,26,29,33,34,35,36,37,38]. In other words, stronger teams have shorter defensive transitions.

The find of this study will allow the coach to know the habitual practices of the high-level teams in this type of situation, to know the factors that possess a major influence on the direct recovery of the possession of the ball, and to know which execution is most adapted to obtaining major probabilities of success. This information supposes a tool for the design of the training of these moves, in order to perfect them and to improve the team’s performance in the competition.

In future studies, it would be interesting to analyze how the ball is recovered (through forced or unforced errors), to include larger samples, and also to compare the same variables in successful and unsuccessful teams. While we are aware of the limitations of our study, we believe that our results will provide information that will be of use to soccer professionals to improve defensive performance.

## Figures and Tables

**Table 1:** Summary of matches analyzed for the study.

Match	Stage	Result	Team/No. of Transitions	
Uruguay–Ghana	Quarter-finals	0–0	Uruguay 49	Ghana 46
Argentina–Germany	Quarter-finals	0–4	Argentina 46	Germany 47
Paraguay–Spain	Quarter-finals	0–1	Paraguay 64	España 54
Netherlands–Brazil	Quarter-finals	2–1	Netherlands 38	Brazil 36
Uruguay–Netherlands	Semi-finals	2–3	Uruguay 60	Netherlands 54
Germany–Spain	Semi-finals	0–1	Germany55	Spain 54
Uruguay–Germany	Third place	2–3	Uruguay 47	Germany 51
Netherlands–Spain	Final	0–1	Netherlands 56	Spain 47
Total no. of defensive transitions				804

**Table 2:** Investigation criteria, categories and codes.

Criteria	Categories
Duration of defensive transition (DJ)	DJ1 (0–15 s)
	DJ2 (16–30 s)
	DJ3 (>30 s)
Loss of possession zone	Defensive zone (DF)
	Defensive midfield (MD)
	Holding midfield (CE)
	Offensive midfield (MO)
	Offensive zone (OF)
Position of players at start of defensive transition (CEII)	Attacking line recovers ball in front of goalkeeper of team being observed (PA)
	Attacking line recovers ball in front of rear line of team being observed (RA)
	Middle line recovers ball in front of middle line of team being observed (RM)
	Attacking line recovers ball in front of middle line of team being observed (MA)
	Middle line recovers ball in front of middle line of team being observed (MM)
	Rear line recovers ball in front of middle line of team being observed (MR)
	Goalkeeper recovers ball in front of attacking line of team being observed (AP)
	Rear line recovers ball in front of attacking line of team being observed (AR)
	Middle line recovers ball in front of attacking line of team being observed (AM)
	Attacking line recovers ball in front of attacking line of opposing team (AA)
Defensive organization of team being observed (ORD)	Organized defense (ORG)
	Circumstantial defense (CIR)
General defensive approach (PTGD)	Expectant (EXP)
	Persistent (PT)
Period of match (T)	Between minute 0 and minute 15 (T-15)
	Between minute 16 and minute 30 (T-30)
	Between minute 31 and end of first half (T-45)
	Between start of second half and minute 60 (T-60)
	Between minute 61 and minute 75 (T-75)
	Between minute 76 and end of second half (T-90)
	Between start of extra time and end of first half of extra time (T-105)
	Between start of second half of extra time and end of extra time (T-120)
Position of defense lines (PS)	Deep (RPL)
	Middle (PL)
	High (AZ)
End of attack zone (ZF)	Defensive zone (FDF)
	Defensive midfield (FMD)
	Holding midfield (FCE)
	Offensive midfield (FMO)
	Offensive zone (FOF)
Position of players at end of defensive transition (CEIF)	Goalkeeper of team being observed ends defensive transition in front of attacking line of opposing team (FPA)
	Rear line of team being observed ends defensive transition in front of attacking line of opposing team (FRA)
	Rear line ends defensive transition in front of middle line of opposing team (FRM)
	Middle line ends defensive transition in front of attacking line of opposing team (FMA)
	Middle line ends defensive transition in front of middle line of opposing team (FMM)
	Middle line ends defensive transition in front of rear line of opposing team (FMR)
	Attacking line ends defensive transition in front of rear line of opposing team (FAR)
	Attacking line ends defensive transition in front of middle line (FAM)
	Attacking line ends defensive transition in front of goalkeeper of opposing team (FAØ)
Match status (RP)	Winning (G)
	Drawing (EP)
	Losing (P)
Outcome of defensive transition (RJ)	Success (EXI)
	Failure (NEXI)

**Table 3:** Absolute and relative frequencies of the categories.

Criteria	Categories	FA	%
Duration of defensive transition (DJ)	0–15 s (DJ1)	533	66.3
	16–30 s (DJ2)	189	23.5
	>30 s (DJ3)	82	10.2
Loss of possession zone	DF	4	0.5
	MD	63	7.8
	CE	173	21.5
	MO	393	48.9
	OF	171	21.3
Position of players at start of defensive transition (CEII)	PA	1	0.1
	RA	28	3.5
	RM	5	0.6
	MA	8	1
	MM	301	37.4
	MR	38	4.7
	AP	33	4.1
	AR	351	43.7
	AM	14	1.7
	AA	2	0.2
Defensive organization of team being observed (ORD)	ORG	794	98.8
	CIR	10	1.2
General defensive approach (PTGD)	EXP	340	42.3
	PT	464	57.7
Period of match (T)	T-15	141	17.5
	T-30	117	14.6
	T-45	105	13.1
	T-60	150	18.7
	T-75	93	11.6
	T-90	149	18.5
	T-105	27	3.4
	T-120	22	2.7
Position of defense lines (PS)	RPL	39	4.9
	PL	156	19.4
	AZ	609	75.7
End of attack zone (ZF)	FDF	230	28.6
	FMD	298	37.1
	FCE	174	21.6
	FMO	80	10.0
	FOF	22	2.7
Position of players at end of defensive transition (CEIF)	FPA	24	3.0
	FRA	389	48.4
	FRM	17	2.1
	FMA	8	1.0
	FMM	258	32.1
	FMR	8	1.0
	FAR	44	5.5
	FAM	8	1.0
Match status (RP)	G	136	16.9
	EP	533	66.3
	P	135	16.8
Outcome of defensive transition (RJ)	EXI	344	42.8
	NEXI	460	57.2

**Table 4:** Results of bivariate analysis.

Variables	χ^2^	*p*
Duration 0–15 s	7.326	0.007
Duration 16–30 s	0.972	0.324
Duration >30 s	8.101	0.004
Loss of possession zone	5.615	0.018
Position of players at start of defensive transition	0.200	0.655
Defensive organization	0.676	0.411
General defensive approach	1.868	0.172
Position of defense lines	8.953	0.003
End of attack zone	8.629	0.003
Position of players at end of defensive transition	8.121	0.004
Match status	2.383	0.123
Period of match	2.296	0.130

**Table 5:** Logistic regression results.

Variables	B	Error	Wald	*p*	Exp(B)
Possession loss zone	−0.059	0.110	0.286	0.593	0.943
Position of defense	−0.316	0.173	3.317	0.069	0.729
Zone in which defensive transition ends	−0.010	0.094	0.011	0.917	0.990
Position of players at end of defensive transition	−0.085	0.045	3.505	0.061	0.919
Duration 0–15 s	−0.208	0.182	1.311	0.022	0.812
Duration >30 s	0.549	0.290	3.588	0.058	1.732
Constant	2.017	0.475	18.042	0.000	7.513
